# Dr. William Wotkyns Seymour

**Published:** 1904-11

**Authors:** 


### OBITUARY.

Dr. William Wotkyns Seymour, of Troy, N. Y., died suddenly at his home, October 18, 1904, aged 51 years. Dr. Seymour was a graduate of Harvard University and received further medical training in Europe, becoming one of the most prominent physicians in the eastern portion of the state. His father, Dr. William P. Seymour, was also a celebrated physician, and the son obtained early advantages through that fact.

Dr. William Wotkyns Seymour was afflicted with gallstone disease some years ago and crossed the ocean to be operated for that condition by Mr. Lawson Tait. He made a complete recovery and had, himself, operated for similar conditions upon many patients. He was surgeon to the Samaritan Hospital, Troy, and a fellow of the American Association of Obstetricians and Gynecologists.

